# Pavement cells and the topology puzzle

**DOI:** 10.1242/dev.157073

**Published:** 2017-12-01

**Authors:** Ross Carter, Yara E. Sánchez-Corrales, Matthew Hartley, Verônica A. Grieneisen, Athanasius F. M. Marée

**Affiliations:** Computational and Systems Biology, John Innes Centre, Norwich NR4 7UH, UK

**Keywords:** Tissue topology, Topological division models, Image analysis, Pavement cells, *Arabidopsis thaliana*, *Drosophila*

## Abstract

D'Arcy Thompson emphasised the importance of surface tension as a potential driving force in establishing cell shape and topology within tissues. Leaf epidermal pavement cells grow into jigsaw-piece shapes, highly deviating from such classical forms. We investigate the topology of developing *Arabidopsis* leaves composed solely of pavement cells. Image analysis of around 50,000 cells reveals a clear and unique topological signature, deviating from previously studied epidermal tissues. This topological distribution is established early during leaf development, already before the typical pavement cell shapes emerge, with topological homeostasis maintained throughout growth and unaltered between division and maturation zones. Simulating graph models, we identify a heuristic cellular division rule that reproduces the observed topology. Our parsimonious model predicts how and when cells effectively place their division plane with respect to their neighbours. We verify the predicted dynamics through *in vivo* tracking of 800 mitotic events, and conclude that the distinct topology is not a direct consequence of the jigsaw piece-like shape of the cells, but rather owes itself to a strongly life history-driven process, with limited impact from cell-surface mechanics.

## Abstract (precis)

**Summary:** Development of the *Arabidopsis* leaf epidermis topology is driven by deceptively simple rules of cell division, independent of surface tension, cell size and, often complex, cell shape.

## INTRODUCTION

**Fig. 1.**
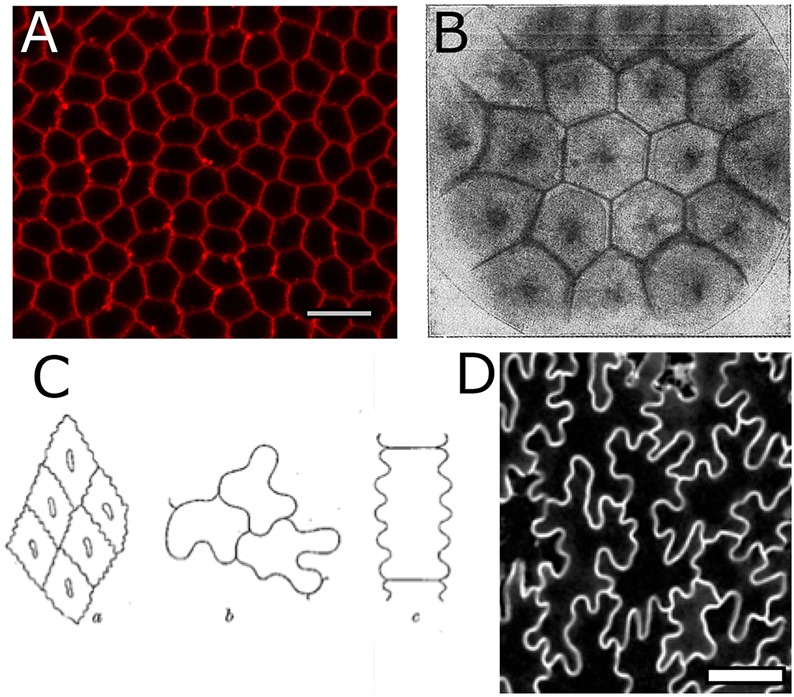
**Foam-like cells and puzzle-like cells.** Biological tissues, such as *Drosophila* epithelium (A), can adopt geometric resemblance to non-biological materials such as ‘artificial tissue’ in which surface tension processes dominate (B), here formed by coloured droplets of a solution diffusing in a less dense solution of the same salt (Fig. 180, p. 501, Thompson, 1942). (C) Cells presenting sinuous outlines (Fig. 186, p. 507, Thompson, 1942): endothelium of a blood-vessel (a); and plant tissues *Impatiens* (b) and *Festuca* (c). (D) Confocal image of the PCs in mature *Arabidopsis* leaves that have grown into jigsaw piece-like shapes. Scale bars: 10 µm in A; 50 µm in D.

Spatiotemporal control of cell growth and division is involved in the generation of tissue shape during development. Tissue shape is likewise affected by biophysical interactions between cells within the tessellated context that modify interfacial lengths and cellular arrangements. Two-dimensional cell layers offer an ideal system in which to investigate the cross-scale processes involved. In *On Growth and Form* D'Arcy Thompson explains how cellular division rules and surface-tension acting upon cells within tissues yield characteristic cell topologies, i.e. specific distributions regarding the number of neighbouring cells, which he regarded as fingerprints of the underlying forces guiding cellular behaviour (Thompson, 1917). Many of his examples refer to biological tissues that resemble foam, with geometries that are strikingly honeycomb-like, such as the *Drosophila* epidermis (Fig. 1A). In cellular materials in which surface tension dominates, cells tend to acquire hexagonal shapes, i.e. six neighbours (‘edges’ in graph theory), even in artificial tissue (Fig. 1B) (Farhadifar et al., 2007; Lecuit and Lenne, 2007; Lewis, 1931; Magno et al., 2015; Thompson, 1917). These regular hexagons minimise surface area for equally sized cells, optimising packing (Durand, 2015; Hales, 2001; Weaire and Rivier, 1984). D'Arcy Thompson also drew attention to a few ‘misfits’ in the cell shape zoo: endothelium of blood-vessels (Fig. 1Ca), epithelial cells of the mussel gills and, finally, epidermal pavement cells (PCs) of plant leaves (Fig. 1Cb,c,D). Their odd sinusoidal features seem to defy the principles of surface minimisation. D'Arcy Thompson offers an explanation through analogy: ʻIf we make a froth of white-of-egg upon a stretched sheet of rubber, the cells of the froth will tend to assume their normal hexagonal pattern; but relax the elastic membrane, and the cell-walls are thrown into beautiful sinuous or wavy folds' (p. 507, Thompson, 1942). He argues that buckling forces could operate in animal epithelia, accounting for sinusoidal cellular interfaces. Yet, for the jigsaw piece-like shape of PCs, he briefly comments: ʻthe more coarsely sinuous outlines of the epithelium in many plants is another story, and not so easily accounted for' (p. 507, Thompson, 1942).

Recent molecular and biophysical studies have confirmed that PC shapes arise due to active internal processes driving anisotropic growth, a consequence of intracellular patterning (Fu et al., 2005, 2009; Gu et al., 2006). The internal patterning involves feedbacks between Rho proteins of plants and cytoskeletal elements (Fu et al., 2005, 2009; Grieneisen, 2009; Grieneisen et al., 2013), modifying structural properties of the cell walls, thereby triggering lobe and indentation formation between those cells (Fu et al., 2009). Essentially, PC lobes present tip-like growth along the convex side, driven by localised actin filaments involved in vesicle transport, as well as other associated proteins, whereas microtubules organise to restrict the concave regions from expanding at a comparable rate (Armour et al., 2015). Interactions between subcellular and supracellular stress and microtubuli organisation further elicit amplifying feedbacks that contribute to PC shape (Sampathkumar et al., 2014). Therefore, PC development is a highly active process, as already inferred by D'Arcy Thompson 100 years ago (Thompson, 1917).

We asked whether this unique cellular morphogenesis also acts uniquely on the tissue topology. Cellular topology arises from the interplay between the way cell division is organised and the biophysical interactions among neighbouring cells. Cell divisions modify topology, whereas biophysical interactions influence topology either directly, by triggering neighbourhood changes, or indirectly, through modifications of cell interface length or cell shape, in turn affecting, in topological terms, the next division plane. Although in plant tissue neighbourhood changes are unlikely, the indirect effects of biophysical interactions can be important. In this context, unlike tissues characterised by hexagonal symmetries, PCs seem not to be driven by surface tension. Given that surface tension-driven processes not only generate a clear fingerprint regarding cellular shapes, but also regarding tissue topology (Farhadifar et al., 2007; Magno et al., 2015), we queried whether PC tissue displays a distinct topological composition. Analysing PC topology thus allows us to assess the relative impact of cell-surface mechanics-driven mechanisms versus life history-driven mechanisms in the establishment of tissue topology, which, in turn, constrains the potential diversity in cell shapes and sizes.

## RESULTS

The *Arabidopsis* leaf epidermis provides an ideal system in which to observe neighbourhood topology within a developing tissue, as it is relatively flat during development and is composed of a single layer of thin (quasi-2D) cells.

### Focusing on pavement cells: from wild type to *spch*

We time-lapse imaged wild-type *Arabidopsis* leaf epidermis (see Material and Methods). The leaf epithelium is composed of different cell types. Intermingled with PCs are stomatal lineages: meristemoids and associated sister cells, guard mother and guard cells (Gray, 2007). The stomatal lineage undergoes tightly organised and regulated divisions to ultimately form stomata (Lau and Bergmann, 2012). Wild-type *Arabidopsis* leaves present a very broad topological distribution (Fig. S1), much broader than generally found for epithelial plant or animal tissue. The topological distribution changes over time. It is, however, difficult to determine why. The particular cell divisions in the stomatal lineage can yield cells with just three or four neighbours, but it is unclear whether this accounts for the overall broadness of the distribution. It is also unclear whether the temporal changes reflect the density increase of the stomatal lineage during this period of leaf development. Alternatively, these temporal changes could be linked to PC dynamics. The division patterns within the stomatal lineage are very different from those of PCs (Asl et al., 2011; MacAlister et al., 2007; Robinson et al., 2011). Both are therefore expected to contribute in distinctive ways to tissue topology. To establish this contribution of each cell lineage requires comparing stomatal lineage cells solely surrounded by cells of the same lineage with PCs solely surrounded by PCs. Two main issues render such an analysis impossible. First, the absence of early meristemoid markers makes it impossible to ascertain whether a cell is a meristemoid, as shape alone is insufficient (Lau and Bergmann, 2012); second, a well-defined separation of contributions cannot be made because almost every cell of the stomatal lineage borders at least one PC, whereas hardly any PC solely neighbours PCs.

Given these difficulties in interpreting wild-type leaf topologies, we proceeded using *Arabidopsis* *speechless* (*spch*) mutant lines (for details, see the Materials and Methods). Mutant plants with no expression of the *SPEECHLESS* gene are unable to produce meristemoids, guard mother cells or stomata (Gray, 2007). *spch* lines allow us to focus on PC tessellations, circumventing the technical and conceptual obstacles indicated above. We followed *spch* leaf growth consecutively over extended periods of up to 15 days.

### Spatial topological patterns over the leaf

As eluded to, topology and geometry are two important aspects to consider when unravelling the mechanisms guiding epithelial development. Geometry refers to the shape and size of the cells, whereas topology refers to their connectivity within the tissue, i.e. the number of neighbours of each cell.

Tissue topology arises due to biophysical processes, which dominate when cell rearrangements are frequent and cell-surface mechanics are prevailing, and to cell division history, which dominates when cell rearrangements are prohibited and cell-surface mechanics plays a limited role in the cell growth and cell shape changes. Here, we focus on the topology of PCs, as our initial hypothesis is that PCs should be strongly skewed towards the regime in which the cell division life history is the dominating factor that guides topology.

**Fig. 2.**
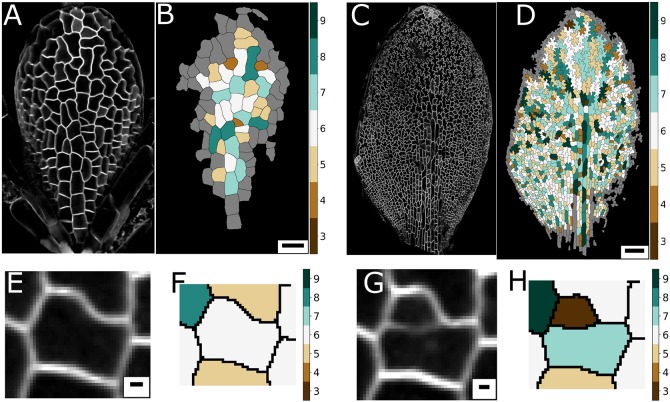
**Topology across the leaf, generated through divisions.** (A-D) The *Arabidopsis* *spch* leaf epidermis at an early time point (A,B), 175.17 hours after stratification (HAS), and a later time point (C,D), 286.50 HAS. (B,D) Heat maps of the neighbour number of each cell for A and C, respectively. (E-H) Divisions influence local topology. Cell before dividing (E), at 220.42 HAS, with neighbour numbers quantified in F. After division, 12.2 h later, the neighbourhood of the cell alters (G), as quantified in H. Scale bars: 20 µm in B; 100 µm in D; 2 µm in E,G.

At early stages of leaf development, for both wild type and *spch*, PC geometry is fairly isotropic (Fig. S1, Fig. 2A,B), with more elongated cells along the midline. As development progresses, cells develop into the ‘jigsaw piece’-like shapes, in a graded fashion from the tip of the leaf to the base (Fig. 2C,D). We first analysed whether the development of such undulating shapes also affects the topology within a PC tissue. To screen for topological patterns at different developmental stages, as well as over the tissue itself, we colour-coded the segmented PCs, indicating for each cell its number of neighbours. Fig. 2B,D presents two distinct time points in leaf development, one early (time point 0, at 175.17 h after stratification) and one later (time point 9, 286.50 h after stratification). We exclude boundary cells or those with an incomplete set of segmented neighbours. A perfectly homogeneous and honeycomb-shaped tissue would appear ‘white’, indicating each cell having six neighbours, see colour bars in Fig. 2. Instead, we find that topology differs from cell to cell. Many cells with fewer than six neighbours, depicted through a brown colour spectrum, and with more than six neighbours, depicted through shades of green, are present in an intermixed fashion, indicating a broad distribution in cell topologies that exist over the leaf. We do not observe a spatial structure in the topology over the tissue, except for larger neighbour numbers along the midvein at later stages, coinciding with cellular elongations in that region.

### Topological distributions are conserved at different developmental stages and within different zones

Within plant tissues, where neighbourhood changes rarely occur (Thompson, 1917), topology is a direct consequence of the previous cell divisions (Mombach et al., 1990). The way cells divide directly affects the topological constitution of their offspring, as well as the topology within their local neighbourhood (Delannay and Le Caër, 1994). This does not imply that biophysics and cell surface mechanics are not involved, as the shapes that cells adopt after division are considered to be important for structuring the next cell division. To illustrate how divisions affect topology, we tracked a particular cell over time, indicating how its division affects the topology of its daughter cells and neighbours (Fig. 2E-H). The mother cell, which originally has six neighbours, generates two daughter cells, with seven and three neighbours. The total number of neighbours of the daughter cells is always *n*+4, independent of *n*, the neighbour number before cell division. Thus, on average they have (*n*/2)+2 neighbours. Consequently, cells with three neighbours tend to gain neighbours; cells with more than four neighbours tend to lose neighbours, more dramatically so for higher neighbour numbers. Note that two neighbours of the mother cell also change topology (Fig. 2E-H). For one cell, the neighbour number increases from six to seven, for the other from five to six. In fact, cell divisions always increase the total number of neighbours in the division neighbourhood by two. Thus, owing to each cell division, the average neighbour number for the neighbouring cells increases by 2/*n*.

**Fig. 3.**
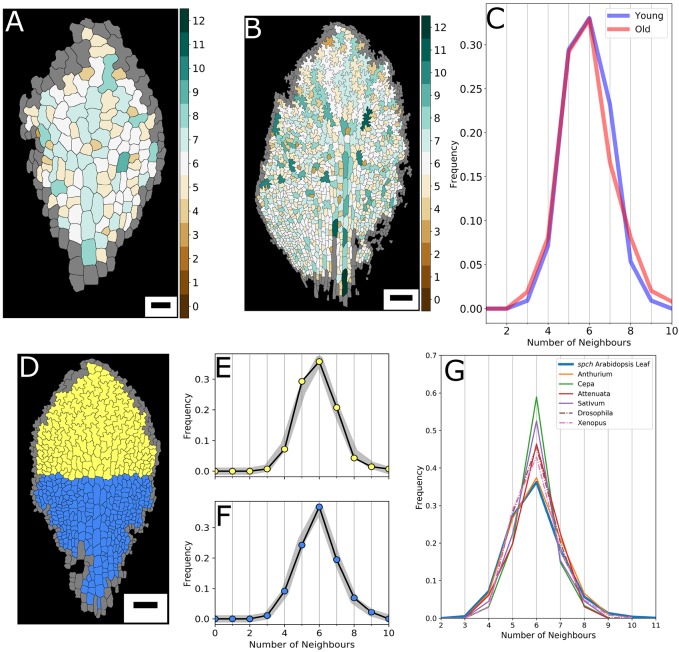
**Topological distributions over time and space.** (A,B) Number of neighbours for each cell for a leaf at 193.25 HAS (A) and at 286.50 HAS (B). (C) Distributions of neighbour frequencies for the ‘young’ (A) and ‘old’ (B) leaf. (D) Meristematic (blue) and differentiation (yellow) zone of a leaf at 232.62 HAS. (E) Topological distribution for the complex-shaped yellow cells, compared with the distribution for that whole leaf (grey). (F) Topological distribution for the blue, less complex and dividing cells, again compared with the whole-leaf distribution (grey). (G) Average topological distributions for animal (broken lines; Gibson et al., 2006) and plant (unbroken lines; Mombach et al., 1990) epidermal tissues from a range of species. The aggregate *spch* dataset consisting of 50,000 PCs presents the least frequent six-sided neighbourhood and most frequent five- and four-sidedness. Scale bars: 20 µm in A; 100 µm in B,D.

We next investigated how topology distributions alter over leaf development, given that cell divisions dynamically change in a temporally and spatially controlled manner (Andriankaja et al., 2012; Asl et al., 2011; Kazama et al., 2010). For a tracked leaf, we compared an early developmental stage, but with sufficient number of cells to allow for meaningful distributions to be made, 193.25 h after stratification (HAS), with a more advanced stage (286.50 HAS). The spatial distributions in neighbour numbers across the tissue (Fig. 3A,B) again do not reveal any noticeable patterning, except for the consistent tendency of higher neighbour numbers at the midvein. Surprisingly, the topological distribution for the entire cell population is unaltered at these different time points, bearing a characteristic profile (Fig. 3C). Thus, the leaf tissue as a whole establishes topological homoeostasis, even though cell geometry changes considerably over these stages, cell numbers still rapidly increase and cell proliferation dramatically varies between different parts of the leaf.

We analysed whether, instead, distinct topological distributions arise in distinct cell populations, by contrasting the topological distribution of the proximal differentiating cells to that of the dividing smaller cells at the base of the leaf (Fig. 3D). This analysis is motivated by our understanding of leaf development: cells divide at a fast rate at the base of the leaf and stop dividing proximally, thereafter mainly expanding and forming complex cell shapes (Andriankaja et al., 2012). Given that the (development of the) topology is directly linked to the cell divisions, an active dividing tissue might present a different topology compared with fully matured tissue. Both populations, however, reveal very similar topological profiles, with a relatively low peak at six and broad ‘shoulders’, including a characteristic skewness to smaller neighbourhood numbers, i.e. a high level of five, and significant fractions of four and three neighbours (Fig. 3E,F).

From the observation that the topological distribution is robust over developmental time and conserved between developmentally distinct zones, we conclude that the number of mitotic rounds cells undergo does not influence the topological distribution. The tissue thus rapidly reaches a topological ‘steady state’, suggesting that the *manner* in which the divisions take place should not depend on developmental time nor on the location within the leaf.

Given that the subsequent cellular development into complex shapes does not impact topology, we asked whether the observed topological distributions therefore resemble those of other plant and animal tissues that do not manifest jigsaw piece-like cell shapes (Fig. 3G). Based upon available published measurements (Gibson et al., 2006; Mombach et al., 1990), the cross-species comparison led to several observations. First, and perhaps not surprisingly, *Arabidopsis* PCs present a topological distribution that is clearly distinct from *Drosophila*. The *Drosophila* imaginal disc is a paradigm epithelial system presenting a ‘surface tension-driven’ topological signature, with roughly equally sized and isotropic cells. Equal tensions between the cell membranes relax cells into hexagonal symmetries (Farhadifar et al., 2007; Lecuit and Lenne, 2007; Sánchez-Gutiérrez et al., 2016), as discussed extensively by Thompson (1942). In fact, all animal epidermal tissues quantified by Gibson et al. (2006), Gibson and Gibson (2009), Li et al. (2012) and Sánchez-Gutiérrez et al. (2016) present a much higher peak of six-sided cells than found in our PC tissue. Moreover, PC tissue is also topologically distinct from other plant epithelia, as reported for *Cepa*, *Sativum* and *Attenuata* (Fig. 3G) (Mombach et al., 1990). In fact, these tissues [as shown by Mombach et al. (1990)] display ordered and staggered brick-like patterning, again resulting in much higher fractions of six-sided cells than found for our PCs. In fact, none of the plant epithelia quantified by Korn and Spalding (1973), Lewis (1928), Mombach et al. (1990), and Sahlin and Jönsson (2010) or animal epithelia quantified by Gibson et al. (2006), Li et al. (2012) and Sánchez-Gutiérrez et al. (2016) present the characteristic PC topology (nor, for that matter, geometries). It is unlikely, however, that the mechanisms driving PC shape formation drive these topological differences directly, given that the PC tissues already present their typical topological distributions prior to the jigsaw piece-like shapes arising (Fig. 3A,F). As an alternative hypothesis, we therefore queried whether the tissue's unique topology could be captured by a set of topological division rules instead.

### Cell division model

**Fig. 4.**
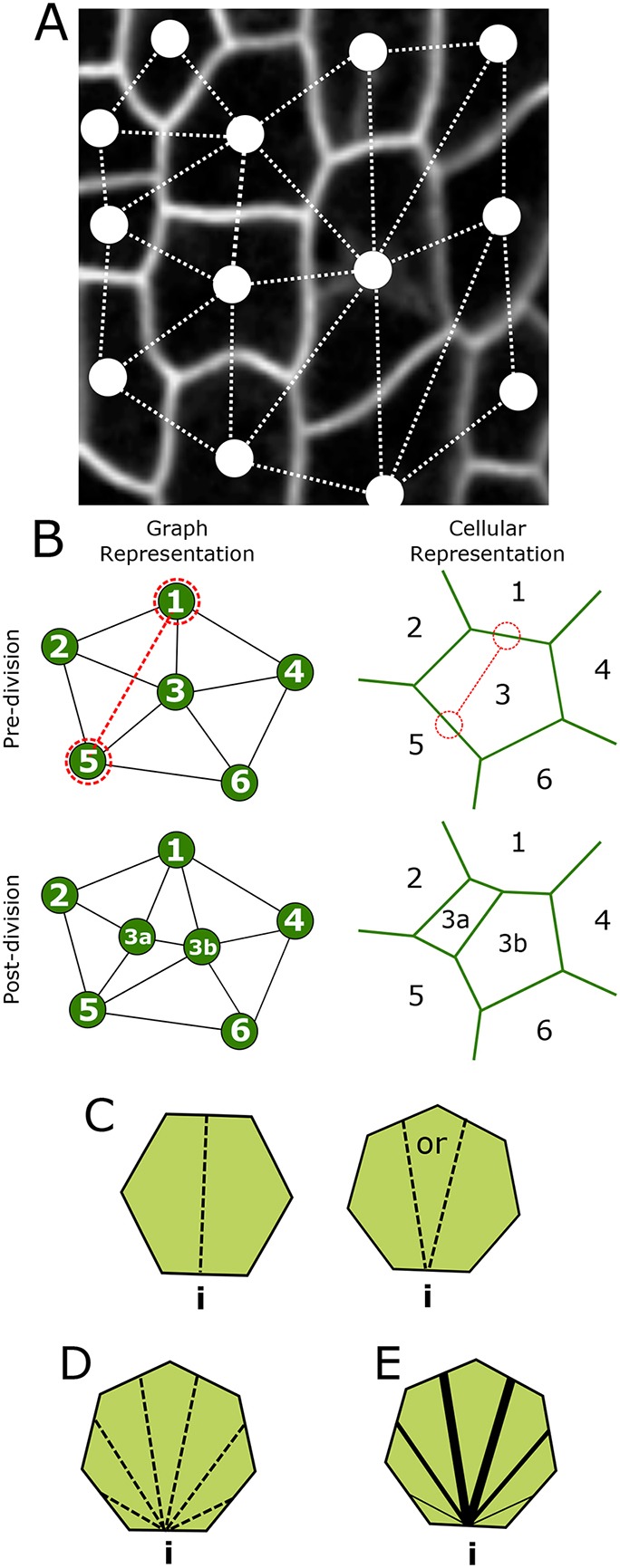
**Graph model for cell divisions.** (A) Network/graph representation superimposed to a cellular representation. Cells are represented by nodes; neighbours by links. (B) Schematic of division implementation within the graph model (left), and its equivalence in space-embedded cellular representation (right). (C-E) The three different division rules, where i represents the initial, randomly selected, wall. (C) ‘Equal split’: neighbours are equally split between the two daughter cells if the cell has an even number of neighbours (left); otherwise, a random choice is made regarding the remaining neighbour (right). (D) ‘Random split’: equal chance for neighbours to be split in any ratio. (E) ‘Pascal split’: neighbour splitting follows a binomial probability, as derived by Gibson et al. (2006), with splits in equal neighbour numbers being more favourable.

We next adopt a purely topological model to determine to what extent the steady state topological distribution of the PCs (Fig. 4A) can be attributed solely to cell division life history. Our representation only describes neighbourhood connections. It explicitly ignores cell shape, therefore serving as a null hypothesis that cell shape and size does not play any role. The epithelium is abstracted to an undirected graph, in which cells are considered to be nodes and links to neighbours are edges. A cellular division, as illustrated in Fig. 4B, adds a cell wall and two daughter cells, increasing the nodes and altering the edges of the graph appropriately. It might seem nonsensical to attempt to capture topological relationships of such an intricate system as the PC tissue through division rules solely based on topological input. This is in stark contrast with the tradition to phrase plant cell division rules in terms of cell shape, e.g. the shortest wall algorithm, Errera's rule, strain-based rules, etc (Sahlin and Jönsson, 2010; Thompson, 1942). We nevertheless simulated several basic topological divisions rules to evaluate to which extent their resulting distributions could, or could not, capture the observed topological distribution.

Simulations take the form of operations on a two-dimensional network. Given that four-way cell junctions are biophysically avoided and mathematically form an infinitely small subset of realised junctions (Thompson, 1942), they are excluded in the model. From this constraint, it follows that the number of neighbours of each cell is equivalent to the number of edges. With such a purely topological description, all that is needed to completely define the process and consequences of cell division, is to specify which cell is dividing and the facets of the two neighbouring cells that are facing the division plane. The division plane is positioned according to the specific topological cell division rule. We consider three possible ways cells can divide, namely an equal split division (Fig. 4C, ‘Equal split’), a randomly oriented division (Fig. 4D, ‘Random split’) and a binomially weighted division (Fig. 4E, ‘Pascal split’). The first scenario, the ‘Equal split’ rule, implies that the new cell wall deterministically forms such as to equally distribute the neighbours of the mother between the daughter cells. After randomly selecting a wall from which the new cell wall emerges, if the number of neighbours is even there is only one possibility for the split (Fig. 4C, left); when uneven, one of the two possibilities is randomly chosen (see Fig. 4C, right). In the ‘Random split’ rule (Fig. 4D), there are no topological pressures whatsoever operating on the choice of the division plane, any combination being equally likely. Finally, the ‘Pascal split’ rule (Fig. 4E) considers it more likely that cells divide so as to equally distribute neighbours between both daughters [using a binomial distribution derived by Gibson et al. (2006)]. The ‘Pascal split’ lies in between the other two rules, as it can asymmetrically distribute the neighbours of the daughters, albeit in a probabilistically decreasing manner. A mechanistic interpretation is that cells divide in two equal parts, the new cell wall connecting two different neighbouring cells, and all other neighbouring cells having an equal and independent likelihood to be adjacent to either one of the newly formed daughter cells (see also Gibson et al., 2006). This is the most likely scenario when cells divide in equal halves, while the interface lengths with the neighbouring cells are randomly distributed. All three division rules assume that the orientation of division is random.

**Fig. 5.**
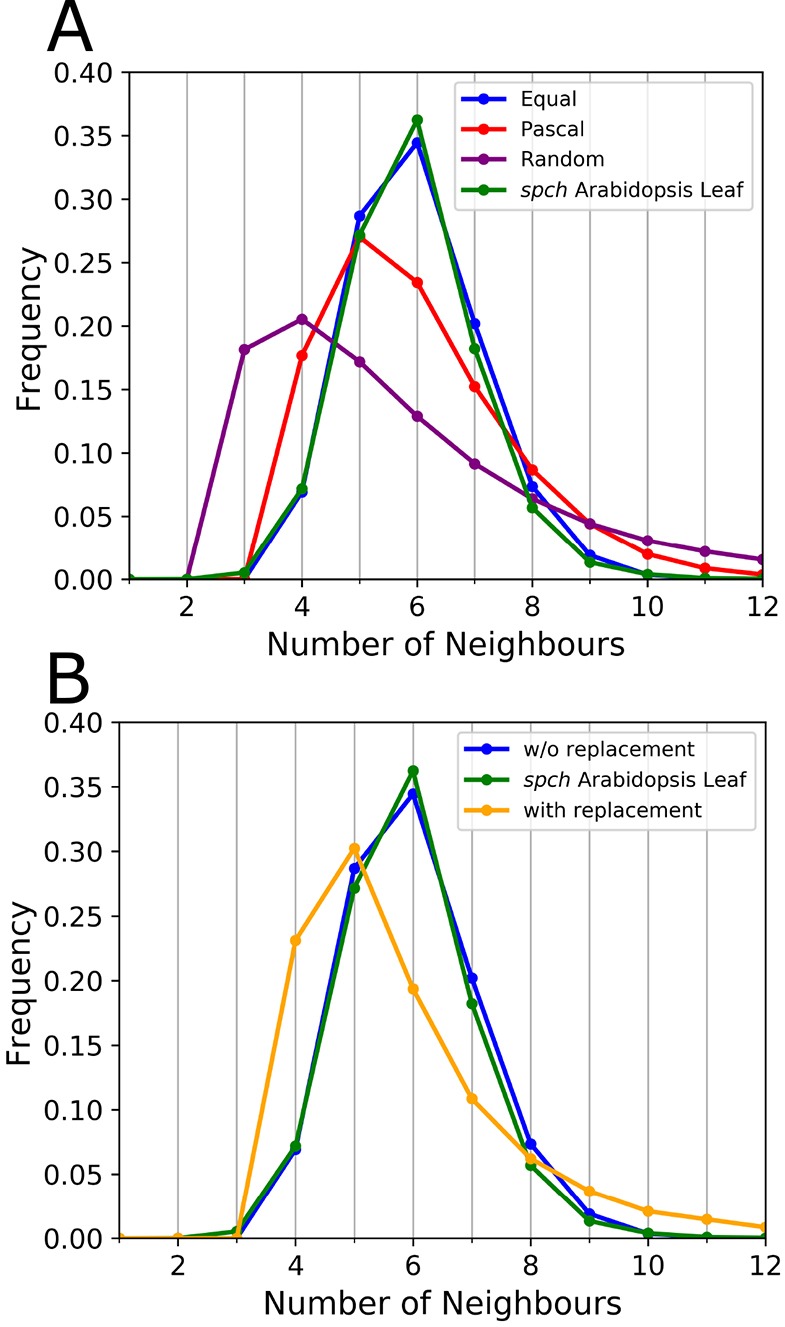
**Topological distributions resulting from underlying rules.** (A) Frequency distributions resulting from the different division rules compared to the aggregated *Arabidopsis* PC tissue data, using the ‘without replacement’ implementation. The mean and the variance, *μ*_2_=< *n*^2^>−<*n*>^2^, of these distributions are *μ*_1_=5.999, *μ*_2_=1.312 (Equal:blue); *μ*_1_=5.999, *μ*_2_=2.695 (Pascal:red); *μ*_1_=5.999, *μ*_2_=10.334 (Random:purple). (B) Distributions resulting from ‘with replacement’ (yellow) and ‘without replacement’ (blue) implementations, compared with aggregate *Arabidopsis* data, both using the ‘Equal split’ rule.

Running iterative rounds of any of these three rules quickly generates steady-state distributions for the final neighbour number frequencies. Surprisingly, our experimental data closely resemble that of the ‘Equal split’ rule, but differs from both the ‘Random’ and ‘Pascal’ rules (Fig. 5A).

There are two main scenarios for these rules within the graph model simulations. In one scenario, each cell (node) performs a single division during each round of divisions of the tissue (graph). This is termed ‘without replacement’. (In statistics, sampling schemes may be without replacement, which means that no element can be selected more than once in the same sample; or with replacement, which means that an element may appear multiple times in the one sample.) Alternatively, the cell to divide is each time randomly selected, its daughter cells have an equal probability of dividing as do all other cells for the next division event. The ‘with replacement’ implementation implies that some cells undergo more divisions than the number of iteration rounds, whereas other cells divide less frequently. In both cases, the divisions are performed asynchronously. Analysing the resultant topological distributions, reveals that the ‘Equal split’ rule ‘without replacement’ closely matches the experimental data, while implemented ‘with replacement’ presents a broader distribution shifted to lower neighbour numbers (Fig. 5B). No other combination reproduces the experimental data, in fact, they all present a lower quality of fit. These results imply that within any local region of the leaf tissue PCs undergo similar division rounds, i.e. the mitotic cycle within local neighbourhoods should be highly comparable. Studies on the shoot apical meristem (SAM) have likewise found that meristematic cells do not simply trigger cell cycle phases upon reaching a critical size, nor that cell division is regulated by a fixed time after birth or through a critical size increment between G2/M transitions (Willis et al., 2016). However, that study also found that cell division behaviour in the SAM was independent of local cell topology and position within the tissue. In contrast, the close match presented between our data and the ‘without replacement’ implementation implies that neighbouring cells undergo similar rounds of divisions, which are then driven by local topology. Please note that our results do not imply that cell divisions are synchronous, but that cells within a local neighbourhood perform a similar number of divisions within a given time window. This is more in line with studies on wild-type *Arabidopsis* leaves that found average cell cycle times to be constant (Asl et al., 2011), and in accordance to how division zones change over time and space (Andriankaja et al., 2012; Kazama et al., 2010). Nevertheless, it is not obvious whether tight control on cell cycle and division zone indeed leads to equal division rounds among neighbouring cells. First, random fluctuations are expected if cells are not actively ‘counting’ their number of divisions. Second, large observed variations in final cell sizes suggest that neighbouring cells do not undergo similar division rounds, as predicted by the topological model.

It is noteworthy that our topological model is well suited to explain PC topological distributions, but is incapable of describing more peaked profiles, such as those of other epithelia (Fig. 2G). This holds for any possible topology-based, cell division history-driven ruleset that does not allow for neighbourhood changes. Any deviation from our ruleset broadens, rather than sharpens, the distribution. This suggests that other tissues, which present more peaked distributions, likely employ a biophysical (or mixed) mechanism to guide their topology and interface rearrangements, which the topological model cannot capture. The PC tissue with its unique topological signature thus represents an extreme example of cell division history-driven topology.

### *In vivo* tracking of underlying topological division relations

**Fig. 6.**
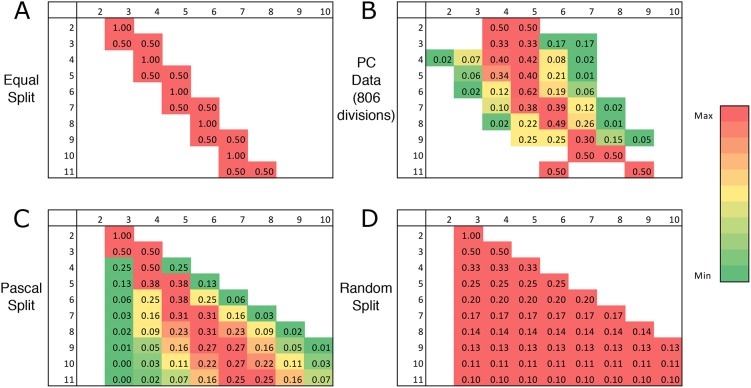
**Comparing theoretical topological division rules with the experimental data.** Division matrices for post-mitotic neighbour number likelihood for the different division rules as well as the experimental data, with the pre-mitotic neighbour number of the cell along the edge and the post-mitotic neighbour number of a daughter cell along the top. The elements indicate the probability that a cell with a given number of neighbours gives rise to a daughter cell with a specific number of neighbours. For visual guidance, green to red colour coding indicates relative likelihood for a given pre-mitotic neighbour number. (A) ‘Equal split’ rule. (B) *Arabidopsis* PC data. (C) ‘Pascal split’ rule. (D) ‘Random split’ rule.

The oversimplification of abstracting cell division behaviour in terms of neighbourhood relations only, raises the issue of how to interpret the surprisingly close match between the resultant profiles. To ascertain the validity of the micro-level assumptions (i.e. how cells position their division plane), we first recall that ‘Equal split without replacement’ provides the best theoretical match of all possible topological rules. However, this does not exclude the possibility that different mechanisms that are not topologically encoded are operating, nevertheless generating a similar tissue-level topology. We therefore followed and analysed the *in vivo* division events themselves (i.e. the ‘micro-level rules’), tracking 806 cell divisions and quantifying the pre- and post-mitotic neighbour number distributions of the mother cell and the resultant daughter cells. This is captured in a matrix relating neighbour number probabilities of the resultant daughter cells to the original neighbour count of the mother cell. Comparing the matrices for the different division rules (Fig. 6A,C,D) with the matrix derived from the *in vivo* tracking (Fig. 6B), we verified that also on the micro-level the ‘Equal split’ rule best resembles the experimental data. Note, however, that this rule is not strictly used in the actual system, as other divisions occur as well at small probabilities. This is visible as non-zero entries in the matrix, mixing elements of ‘Equal split’ and ‘Pascal’ rule. This can partially be explained by image acquisition time intervals being too long to exclude divisions of cells neighbouring the dividing cell. Such divisions can be observed in Fig. 6B through non-zero entries that are impermissible by division of a single cell (such as a three-edged cell giving rise to a daughter cell with more than four neighbours). The experimentally derived matrix is therefore broader than the *de facto* division matrix, expected to be more similar to the ‘Equal split’ matrix. Other deviations stem from rare division events. For example, rows 3 and 11 are based on a single observed mitotic event only. Considering these additional spreads in the experimentally derived matrix, we conclude that PC divisions are well described by the ‘Equal split’ matrix.

### Breaking the law: from Aboav-Weaire back to Lewis

The observations that (1) PC topology can arise from simple topological rules; and (2) the division events are similar to the topological divisions as implemented in the model, prompt the question what role, if any, does cell geometry play? We start probing potential additional regulatory processes involved in the topological outcome by comparing the topological properties of PCs with those of non-biological cellular material.

**Fig. 7.**
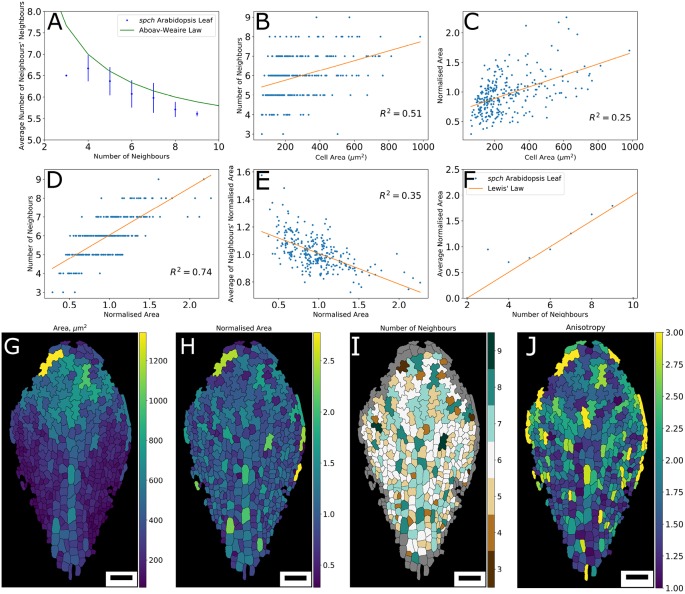
**Relating topology and size.** (A-F) Plots linking topological relationships and cell size for cells of a leaf imaged 220.42 HAS. (A) Number of neighbours versus average number of neighbours' neighbours, with Aboav-Weaire's Law superimposed (green line). (B) Cell area versus number of neighbours. (C) Cell area versus normalised area (cell area/average of neighbours' cell area). (D) Normalised area versus number of neighbours. (E) Normalised area versus average of normalised area of neighbours (excluding the central cell itself). (F) Average normalised area of cells with *n* neighbours against neighbour number, with Lewis' Law superimposed (orange line). (G-J) Heat maps of cell shape properties over the leaf, showing (G) cell area; (H) cell area normalised to average of neighbours; (I) number of neighbours; and (J) anisotropy (major axis/minor axis). Colour bar shown on the right of each image indicates respective cell-level quantities. Linear regression line shown in orange (B-E), with corresponding *R*^2^ value indicated within each panel. Scale bars: 50 µm in G-J.

We do so by analysing, as a null-hypothesis, if the Aboav-Weaire (AW) law holds for our PC data. AW describes a generic, quantitative empirical observation valid for a wide set of (biological and non-biological) cellular materials (Mombach et al., 1990). It is based on the observation that few-edged cells have a remarkable tendency to be in contact with many-edged cells, and vice versa. In its most approximate form, Aboav (1970) found, for a range of non-biological cellular and granular materials, that:
(1)
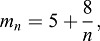
where *m_n_* is the average number of edges (neighbours) of a randomly chosen cell neighbouring a cell with *n* edges (see also Chiu, 1995). Plotting *m_n_* against *n*, our data obey the general trend that cells with higher number of neighbours are surround by cells with, on average, fewer neighbours, in accordance with Eqn 1 (Fig. 7A). However, AW Law consistently overestimates this average and moreover qualitatively diverges from the PC data at *n*=3. The original observations for which the law was derived were not made in a biological context (Aboav, 1970). It has led to several physical theories on how basic entropic considerations can generate such a generic power law (Chiu, 1995; de Almeida and Iglesias, 1988; Peshkin et al., 1991). This yields an important search image: if a biological topological distribution follows AW, as manifested and expected in a physical context, then the explanation might not be ingrained in biological processes, but in considerations stemming from statistical mechanics. However, if the distribution diverges, it indicates that other processes are operating on the system, likely of biological origin.

When analysing the same relationship for our topological models (Fig. S2), we find that they also deviate from AW. The experimental data again most closely matches the ‘Equal split’ rule. Nevertheless, unlike our previous results, we now observe a clear discrepancy between the experimental data and the topological model. The experimental data show a more pronounced relationship between neighbour number and the number of neighbours of the neighbours, indicating that the mechanisms underlying the second-order neighbourhood topology cannot be captured by a basic topological rule.

One way to intuitively interpret the experimentally observed relationship is that having more neighbours is directly linked to covering a larger area, while these neighbours cover on average smaller areas, hence having fewer neighbours themselves. Indeed, Peshkin et al. (1991) have derived AW using the maximal entropy principal, arguing that, from a statistical perspective, larger cells tend to neighbour, on average, smaller ones. To verify the first assumption for our experimental data, i.e. that having more neighbours is linked to being larger, we plotted the number of neighbours against cell size, but only roughly found this basic trend (Fig. 7B). The graph is very scattered (*R*^2^=0.51). The gradual increase in size over the PC tissue (Fig. 7H) invalidates the first part of the explanation.

Realising that the large variations in cell area over the tissue obfuscate this relationship, we next plotted the ratio of cell area over the average cell area of neighbours (termed ‘normalised area’), against absolute cell area (Fig. 7C). This again does not produce a strong correlation, although big cells do tend to be bigger than their neighbours, and small cells tend to be smaller than their neighbours. If, however, neighbour number is plotted against normalised area (Fig. 7D), a much stronger correlation emerges. This means that within the PC tissue a cell that is relatively big in comparison with its neighbours has, on average, more neighbours. Conversely, being smaller than its neighbours, a cell tends to have fewer neighbours. Although this relationship might seem trivial, it can easily be overlooked in plant tissues, as it requires local area normalisation. To test the second assumption, i.e. that larger cells are surrounded by smaller cells, we plotted the average of the normalised areas of the neighbours (i.e. the relative size of the neighbours in regard to their own neighbours) against the normalised area of the given cell (Fig. 7E). To prevent circular reasoning (i.e. my neighbour is smaller than me because I am larger than my neighbour), the central cell was excluded when determining the normalised areas of the neighbours. Nevertheless, we found a clear relationship supporting the assumption that cells surrounding larger cells are truly smaller than average. This relationship presents stronger correlation than the normalised area versus area relationship (compare Fig. 7C with Fig. 7E). Although those two observations together can explain the AW trend in Fig. 7A, the underlying mechanism driving the local cell size variation remains unclear.

Further linking topology to cell geometry, Lewis' Law empirically relates the number of neighbours with the average area of cells of that topological category (Lewis, 1928):
(2)
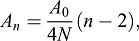
where *n* represents the number of neighbours of a cell; *A_n_* the average area of cells with *n* neighbours; *N* the total number of cells; and *A_0_* the total tissue area. Relating our data in a similar manner using normalised areas reveals that Eqn 2 indeed holds for larger *n* values, but deviates substantially at lower topologies, such as *n*=4 and 3 (Fig. 7F). Lewis' Law can be regarded as the consequence of an equilibrium between entropy and organised form in cellular material. It is a direct consequence of the existence of space-filling cells and their topology. Rivier and Lissowski (1982) derived formally, using statistical mechanical considerations, that this law corresponds to the maximal arbitrariness in the distribution of topological categories of cells that compose a 2D tessellated structure. Their result implies that if a tissue does not follow this relationship (Eqn 2), such as is the case for *n*=3,4 in our PC tissue, then the average cell area is not simply regulated by the area-filling requirement, but instead other biophysical constraints or biological processes are involved (as also recently shown by Kim et al., 2014). Based on these topological and geometrical considerations, we conclude that the deviations of the PC data from both AW and Lewis' Law point to the existence of additional regulatory dynamics operating during PC development within the leaf. We propose that they are most likely linked to the division dynamics in conjunction with cell shape mechanics.

## DISCUSSION

We revisited theories put forward by D'Arcy Thompson, armed with novel mathematical and computational approaches and unprecedented potential for data analysis. By analysing a mutant *Arabidopsis* line composed only of PCs, we could focus on the interactions between a similar population of large, non-surface-tension-minimising cells. It allowed us to bypass the natural heterogeneity in patterning and tessellation that is present in a wild-type leaf.

Using advanced microscopy techniques, we could accompany the growth of several leaves, and follow the development of the individual cells, tracking roughly 50,000 cells and more than 800 cell division events, automatically capturing their topological properties. We found that, despite the characteristic shapes these cells acquire, the topological distribution is conserved between the population of dividing and differentiating cells. Thus, a topological steady state is reached prior to the PC shape transformation. Despite the topology being shape and time independent, the distribution was unique when compared with other systems: PC tissue consistently presents less six-sided and more five-sided cells than any other animal or plant tissue studied thus far (Gibson et al., 2006; Korn and Spalding, 1973; Mombach et al., 1990; Sahlin and Jönsson, 2010).

A large body of theoretical work has focused on investigating the physical and statistical basis that underpins the topological distributions of non-biological and biological materials (Delannay and Le Caër, 1994; Dubertret and Rivier, 1997; Durand, 2015; Durand et al., 2014). Relevant in our context is the insight that when it is possible to fix the peak of the topological distribution to *n*=6 while the extra degrees of freedom allow the alteration in the variance of the distribution (*μ*_2_=< *n*^2^>−<*n*>^2^), a useful relationship can be derived between the frequency of six-sided cells, *P*(6), and the variance of the entire frequency distribution, *μ*_2_, by means of assuming a poisson distribution for the possible cellular topologies within the tissue (Le Caër and Delannay, 1993). The (approximate) relationship derived and validated by Le Caër and Delannay (1993) is as follows:
(3)
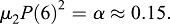
In accordance with this predicted relationship, our experimental data generates *α*=0.150049, while the topological simulation employing ‘Equal split without replacement’ yields *α*=0.1558. This further confirms that the relationship between the average frequency of six-sided cells and the spread of the topological distribution can be captured semi-universally by a single parameter. Further constraining such relationships, Durand et al. (2011) more recently developed analytical models for the statistical mechanics of shuffled two-dimensional cellular tissue to reveal a strict correlation, without any adjustable parameter, between topology and geometry. Their work shows that the standard deviations in the frequency distribution of *n*-sided cells (*μ*_2_) and in the cell areas themselves (Δ*A*) are in proportion (Durand et al., 2011). In *Drosophila*, this link between areal and topological variations was experimentally verified and further corroborated through Voronoi models (Sánchez-Gutiérrez et al., 2016). We therefore queried whether the topological distribution of the PCs could likewise be linked to the areal variation. This, however, was not the case, challenging the affirmation that variation in topology can be correlated unequivocally to areal variation. It follows straightforwardly from the observation that PC topology frequency distributions are preserved at different time points (Fig. 3A-C), whereas relative areal variation varies considerably between those time points (Fig. S3A-C). A same observation can be made when comparing the distinct dividing and differentiating cell populations (compare Fig. 3D-F with Fig. S3D,E). This is indicative that for our system a memory-based explanation (Li et al., 2012), which relies mainly on cell division events, and with non-rigorous constraints on cell surface mechanics, is most appropriate.

Corroborating with this, our topological model, based on graph simulations, showed that the observed topological PC distributions could be reproduced by a population of cells that undergo similar rounds of divisions and in a manner that equalises the number of neighbours between the two daughter cells. Accordingly, on the level of the rules which generate these statistical distributions, we find a match between the division events themselves: the experimental data shows a similar trend in the topological redistributions that govern their division planes.

Although this parsimonious model captures surprisingly well the macroscopic and microscopic events within the PC tissue, it is not straightforward to biologically interpret these results. What does such a model agreement imply regarding the mechanisms that cells use to make the relevant cell division decisions? A naive and direct interpretation is that mechanisms are in place for cells to directly assess the number and distribution of neighbours, in such a way that division planes are laid down to equally distribute the neighbours among the newly formed daughter cells. It could be that the required topological information is directly exerted through mechanical transduction of the tricellular junctions, such that the positioning of the new cell wall is a function of the tricellular junction distribution only. We refer to such a model, which is cell-interface independent (and thus essentially different from cell surface mechanics), as a ‘tent model’. Strains within camping tents are greatly exerted by the pegs that secure the tent down and strain the network of poles. Similar concepts of internal network force distributions have been proposed for animal cells, through tensegrity models (Ingber, 2004), while a recent study on *Drosophila* epithelium suggests direct tricellular junction detection (Bosveld et al., 2016). In addition, neighbours could also be perceived in non-mechanical ways, e.g. through plasmodesmata-mediated cell-cell communication.

Nevertheless, we do not consider it immediately helpful to interpret our topological model in such a literal manner. The tent model, albeit offering a potential mechanistic basis for how topology could be sensed, poses a distraction from the possibility that the topological division rule is a side-effect or proxy at a higher level of description capturing the consequences of underlying division and growth mechanisms combined. The model presented here does not disqualify geometrical or tissue tension models (Besson and Dumais, 2011; Louveaux et al., 2016), but rather raises questions as to how such different views at different levels, geometry and topology, can be reconciled.

Such a debate is analogous to one in the field of developmental plant modelling, where two important classes of auxin transport models, ‘up the gradient’ and ‘with the flux’ parsimoniously capture behaviours of tissue polarity in relation to PINs (auxin efflux carriers), but are based on assumptions that do not need to be interpreted biologically in the same manner as they are encoded (Grieneisen and Scheres, 2009). Recent research efforts show entirely other molecular mechanisms underlying rules that lead to ‘up the gradient’ and ‘with the flux’ descriptions (Abley et al., 2013, 2016; Cieslak et al., 2015). A similar view can be adopted for interpreting topological rules: either they are generated directly through the tent model or intricate cell-cell signalling; or they emerge from lower-level mechanisms on the basis of cell geometry and polarity, yet mimicking the topological behaviour.

With regard to geometrical considerations, Besson and Dumais (2011) generalised Errera's rule (Errera, 1886) for cell division, correctly predicting for a wide set of plant species (including ferns and green algae) the position and shape of the division plane by considering energy minimisation ‘alike a soap bubble’ (for an in-depth analysis of Errera's conjecture, see Thompson, 1942). Although their work does not describe how iterations of such divisions affect topology, our topological approach does not at all consider cell shape. Could their rule hold for PCs and explain its unique topological distribution? Using the calculus of variations as presented by Besson and Dumais (2011) to test the generalised Errera's hypothesis is prohibitively cumbersome for PC shapes, owing to the required numerical exploration of the highly complex configuration space involved. However, it has been shown that the Besson-Dumais rule fails to account for cell division plane orientation when growth becomes heterogeneous and tissue curvature becomes anisotropic (Louveaux et al., 2016). As these characteristics are present in the leaf epidermis, this rule should not be able to capture PC divisions. Moreover, PC divisions do not result in equally sized cells (Fig. S5), contradicting an important additional constraint typically used when applying Errera's rule (Besson and Dumais, 2011). Nevertheless, it is interesting to consider how geometric constraints might be adapted within such an approach to account for the PC divisions.

Indeed, significant attention has recently been given to how cells assess their shapes and sizes (Chen et al., 1997; Li et al., 2012; Willis et al., 2016), and use such geometric inputs to guide their division planes. What is not so clear, though, is how biological cellular behaviour driven by shape generates on the topological level the behaviour that we find describes so well the data. Based merely on cell sizes (Fig. 7A-F), our data and analysis suggest that purely topological and entropic effects cannot fully explain all observed relationships. For example, the deviations from Lewis' Law indicate that other mechanisms, of biological origin, are operating. Comparing the PC topological distribution with computer-simulated distributions based on shape-dependent division rules as analysed by Sahlin and Jönsson (2010), we also found, among the very divergent profiles, one clear match (Fig. S4). In that work, computer simulations were performed based on a vertex model of equally sized cells with anisotropic growth, including surface-tension driven processes acting on the cells to rearrange the distances between vertexes. Their spatially embedded cellular model prohibits neighbourhood swaps (i.e. T1s), an important assumption when addressing topological changes through division in plant tissues. Contrasting our topological distributions with those generated by their different division hypotheses revealed a large qualitative spread among their results, as well as a large divergence between their experimental *Arabidopsis* SAM data and our PC tissue data (Fig. S4A). The rule that generated the most closely resembling topological distribution is the ‘Random split through the centre of mass’ rule (Fig. S4B). The close correspondence between these distinct implementations of cell divisions suggests that, under isotropic growth, surface tension processes coupled with cell growth effectively redistribute cell edges along each individual cell such that when divisions occur through the centre of mass and in a random direction, this new cell wall tends to split the cell into two equal parts, effectively distributing the neighbours equally between the daughter cells, as performed by our graph model.

However, *Arabidopsis* leaves do not grow isotropically (Donnelly et al., 1999; Kuchen et al., 2012), nor are the cells in *spch* of equal size or anisotropy (Fig. 7G,H,J). It therefore remains an unresolved issue which shape-dependent rules – if any – can be mapped onto the topological rules we find here when the complex growth patterns of the *Arabidopsis* leaf are fully taken into account. Furthermore, future studies are needed to dive deeper into the control mechanisms at the molecular level that can account for the observed cell division behaviour that currently at least phenomenologically explains the topological distributions. Thus, the quest for mechanisms linking passive biophysics to active cell behaviour based on shape, size and topology – as formalised and initiated by D'Arcy Thompson a century ago – has still to be finalised, as necessary today as it was then, to unravel how tissues develop their characteristic and unique properties during growth.

## MATERIALS AND METHODS

### Confocal images and image processing

We analysed both wild-type and *speechless* (*spch4*) (MacAlister et al., 2007) leaves. Both were crossed with a membrane marker, pmCherry-Aquaporin (Nelson et al., 2007), to visualise the boundaries between cells during confocal microscopy. Plants were grown and imaged in a custom-built perfusion chamber (growth chamber) (Calder et al., 2015; Kuchen et al., 2012; Robinson et al., 2011; Sauret-Güeto et al., 2012), from around 7 to 23 days after stratification. Within the experimental growth chamber setting, *spch* leaves grow similarly enough to wild type to justify their use as a model system (Kuchen et al., 2012; Sánchez-Corrales, 2014). For this study, one wild-type and seven *spch* leaves were imaged at 15 time points for the wild-type leaf and 121 time points for the *spch* leaves. Confocal stacks were projected and segmented using custom software and segmented cells were matched between successive time points. The data are in the form of segmented images, where cell colour corresponds to a unique cell ID. The automatic pipeline can cause over-segmentation. Segmented data has therefore been manually hand-curated by means of a careful visual check. After segmentation correction, tracking is performed, which links the cell IDs between segmented images. The tracking has also been visually checked and hand-curated for any possible errors. To avoid introducing artefacts to the statistical topological analysis, we consider only cells with a fully defined neighbourhood that are not located at an edge of the segmentation (grey cells).

### Cell-division model

Simulations take the form of operations on a network. An epithelial tissue can be abstracted to an undirected graph, where cells are considered as nodes and links to neighbours are considered as edges. By assuming the tissue has no four-way cell junctions, the number of neighbours a cell has is equivalent to the number of sides it has. With this formalism, all that is needed to completely define a cell division is the cell that is dividing and the two neighbouring cells between which the division plane is placed. The positioning of the division plane depends upon the desired cell division behaviour. For the purposes of this study, we considered three different types of behaviour: a randomly oriented division plane, a binomially weighted division plane and an equal split division plane. These three behaviours give different steady-state distributions for the final neighbour number frequencies and our study is concerned with which behaviour most closely matches our data.

## Supplementary Material

Supplementary information

Supplementary information
