# Supplementary material for: Pavement cells and the topology puzzle
Source: Development. 2017 Dec 1;144(23):4386–97. doi: 10.1242/dev.157073 (PMC5769637; doi:10.1242/dev.157073)
Supplement: Supplementary information [file develop-144-157073-s1.pdf]

Supplementary Material for

Pavement cells and the topology puzzle

Ross Carter[1,*], Yara E. Sánchez-Corrales[1,2,*], Matthew Hartley[1], Verônica A. Grieneisen[1,†] and Athanasius F. M. Marée[1,†]

[1]Computational and Systems Biology, John Innes Centre, Norwich NR4 7UH, UK.

[2]Current address: MRC-Laboratory of Molecular Biology, Cambridge Biomedical Campus, Francis Crick Avenue, Cambridge CB2 0QH, UK.

[*]These authors contributed equally to this work.

[†]To whom correspondence should be addressed.
E-mail: Veronica.Grieneisen@jic.ac.uk, Stan.Maree@jic.ac.uk

Supporting Figures

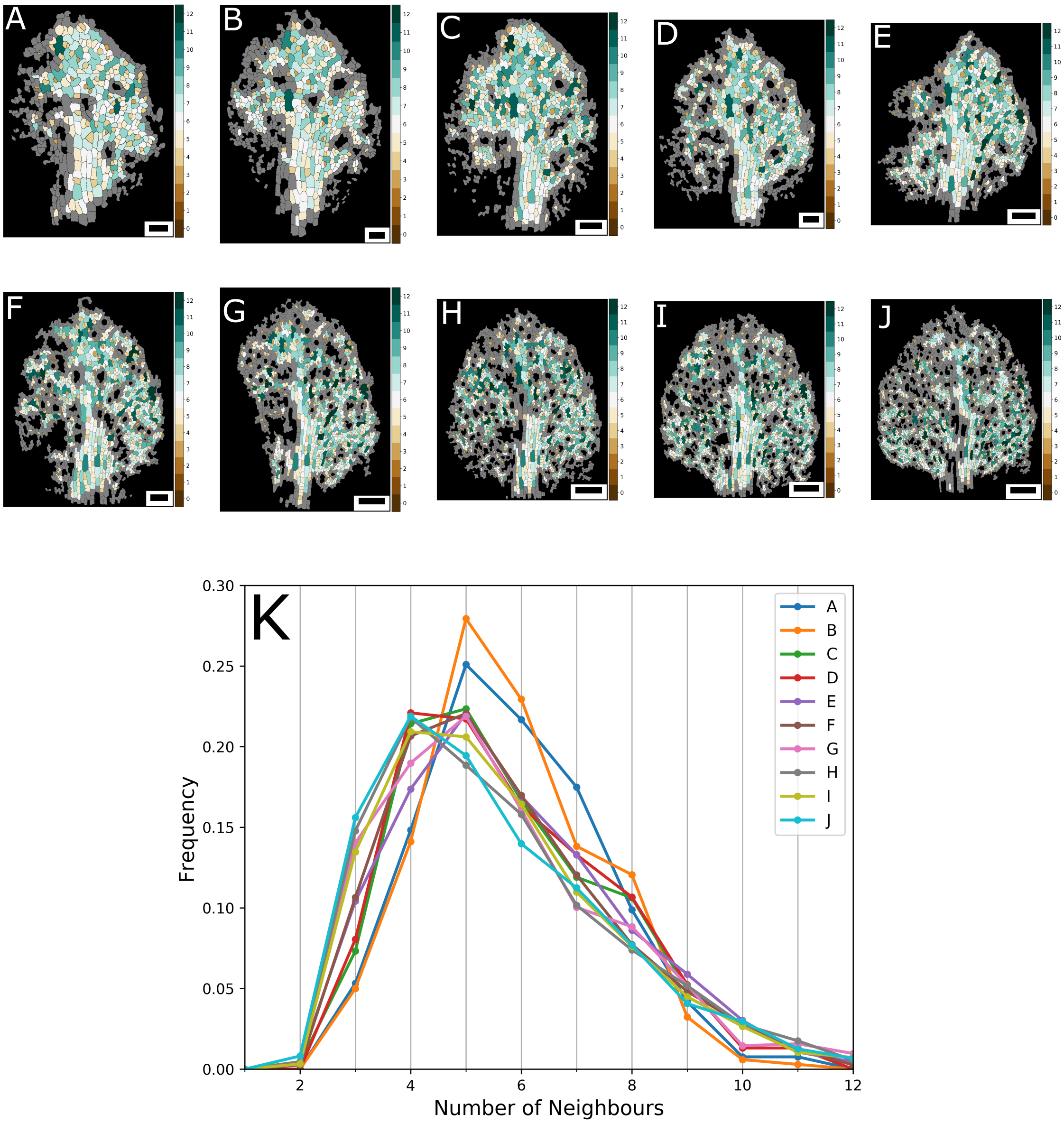

Figure S1: **Neighbourhood distributions in WT leaves.** (A–J) Heat maps of the number of neighbours of each cell, for time-points between 144 and 185.5 HAS. (K) Neighbourhood distributions for the leaves shown in (A–J). Time-points: (A) 144 HAS; (B) 148.5 HAS; (C) 156.5 HAS; (D) 160.5 HAS; (E) 164.5 HAS; (F) 168.5 HAS; (G) 172.75 HAS; (H) 176.75 HAS; (I) 180.75 HAS; (J) 185.5 HAS. Scale bars: (A, B) 20 μm; (C, D) 50 μm; (E, F) 100 μm; (G–J) 200 μm.

Development • Supplementary information

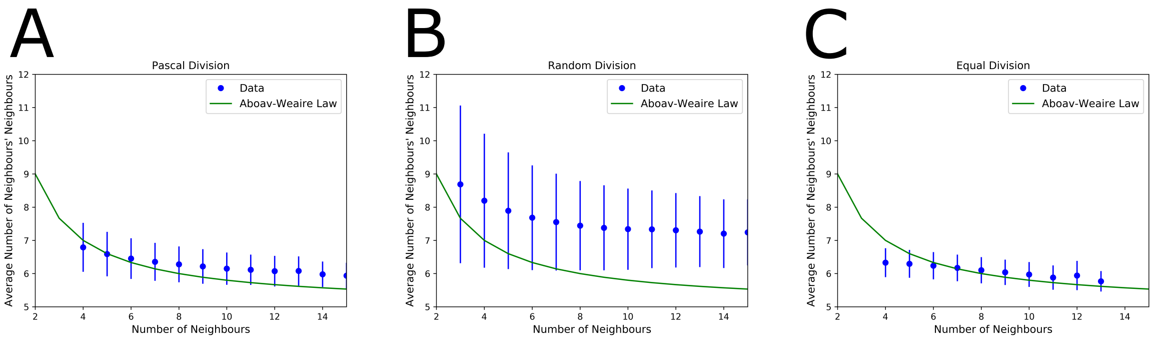

Figure S2: **Predicted number of neighbours' neighbours in the topology model and Aboav-Weaire's Law.** Average number of and standard deviation in neighbours' neighbours, as a function of neighbour number, for the different topological division rules. (A) 'Pascal split'; (B) 'Random split'; and (C) 'Equal split' rule.

Development • Supplementary information

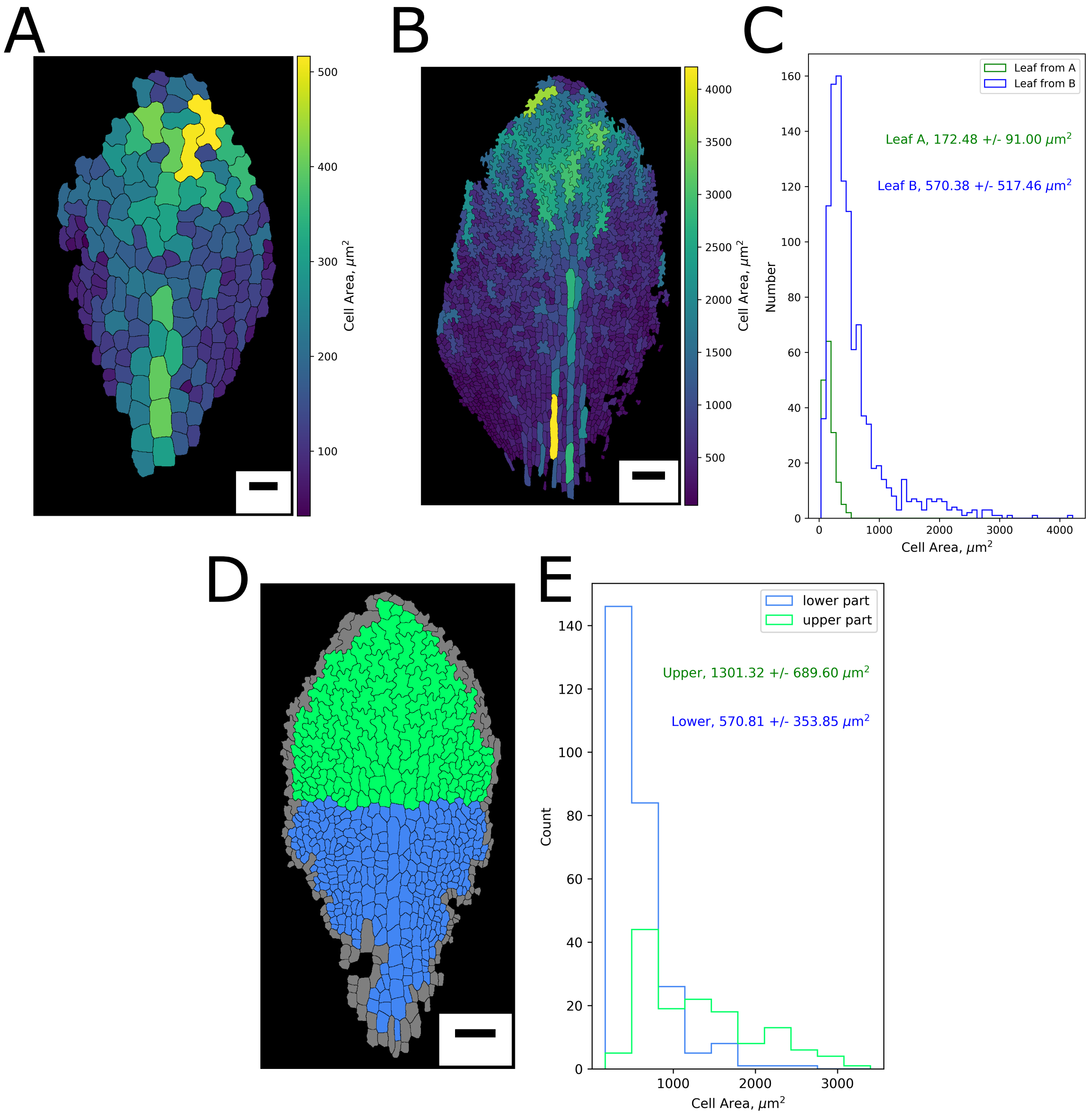

Figure S3: **Variation in cellular area over the tissue.** (A) Spatial distribution of cellular areas, shown through colour bar to the right of the panel, of a young leaf (93.25 HAS). (B) Spatial distribution of cellular areas of a more mature leaf (286.50 HAS). Both tissues correspond to Figure 3A, B. (C) Plot comparing both area distributions, indicating average area and standard deviation. (D) Population of dividing (blue) and differentiating (green) cells, as also shown in Figure 3D. (E) Area distributions of these distinct populations, indicating average area and standard deviation. Scale bars: (A) 20 μm; (B, D) 100 μm.

Development • Supplementary information

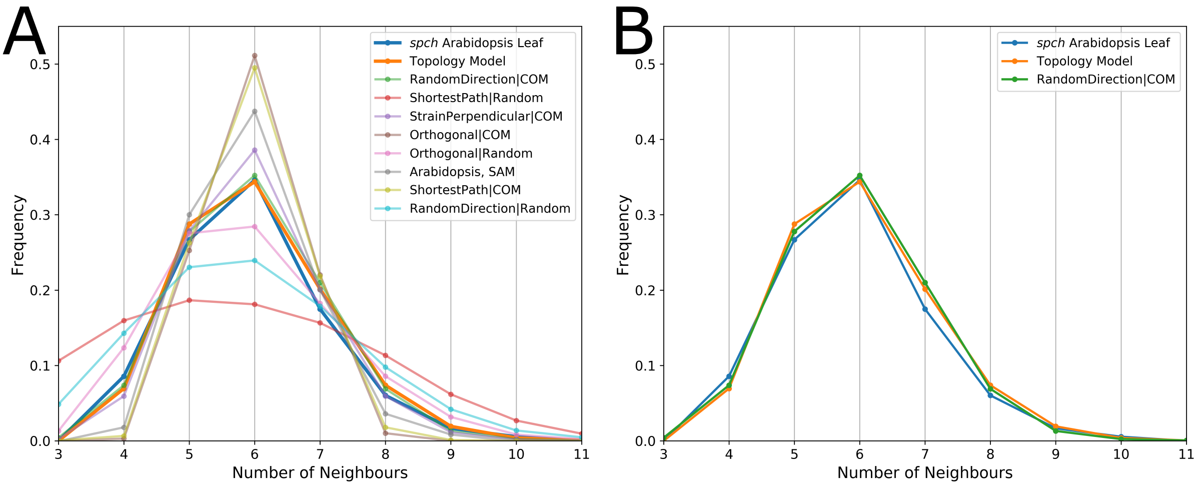

Figure S4: **Geometric division-rules compared to PC data.** (A) Topological distributions as generated by the division rules of the vertex-based model presented in Sahlin and Jönsson (2010), compared to the distributions generated by our topological model and observed in our experimental PC data. (B) Only the Random Direction through Centre of Mass rule bears close similarity to our topological 'Equal split' rule and PC data.

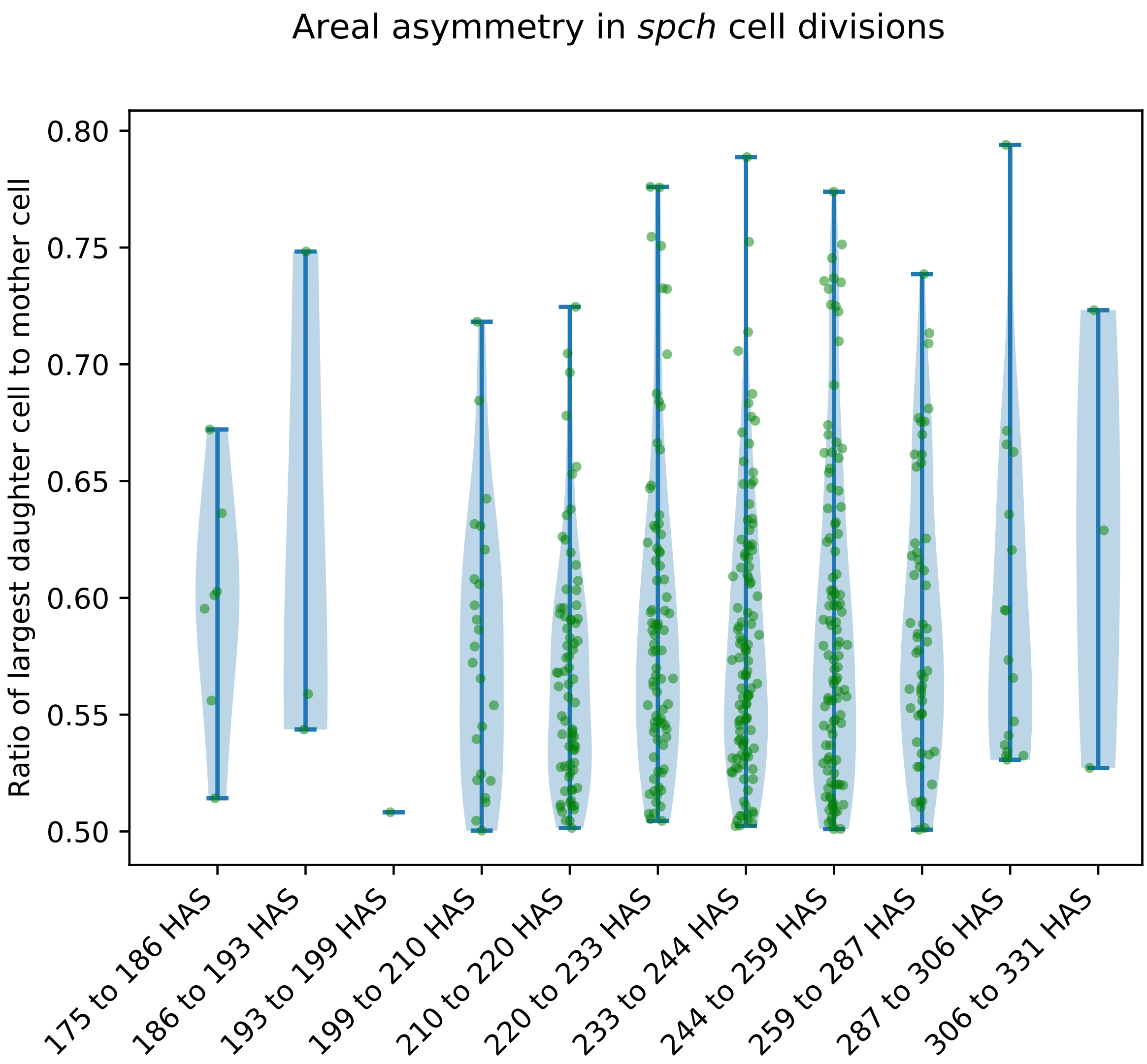

Figure S5: **Pavement cell divisions are not restricted in generating equally sized daughter cells.** Data of one tracked *spch* leaf. Each green point represents a mitotic event during a specific time-interval of the leaf development, as indicated along the x-axis. The value along the y-axis indicates the ratio between the largest daughter cell and its mother cell. Because of the areal growth during each time interval, we do not directly use the area of the mother cell before division, but instead calculate it as the summed area of the two daughter cells. If cells were to divide in a manner to generate equally sized cells, this value would be 0.5. Larger values indicate the magnitude of asymmetry in these divisions. Blue shaded regions depict distributions of variations.
